# Currents in a Quantum Nanoring Controlled by Non-Classical Electromagnetic Field

**DOI:** 10.3390/e23060652

**Published:** 2021-05-23

**Authors:** Jerzy Dajka

**Affiliations:** 1 Institute of Physics, University of Silesia in Katowice, 40-007 Katowice, Poland; jerzy.dajka@us.edu.pl; 2 Silesian Center for Education and Interdisciplinary Research, University of Silesia in Katowice, 41-500 Chorzów, Poland

**Keywords:** quantum ring, entanglement, non-classical electromagnetic field, persistent current

## Abstract

Quantum ring accommodating interacting spin-less fermions threaded by magnetic flux with a non-classical component added to a static, inducing persistent current, is considered. It is investigated how current flowing in the ring becomes affected by a state of non-classical flux and how Coulomb interaction between fermions influences entanglement of quantum ring and the driving field. In particular it is shown that in an absence of decoherence and under certain conditions fermion–fermion interaction is necessary for a ring–field entanglement to occur.

## 1. Introduction

Quantum information processing [1] meets nanoscience in applications related both to quantum computing and communication. Superconducting devices [2,3] utilizing Josephson behaviour as a cornerstone [4,5,6] are very often building blocks of many of these applications. Furthermore, non-superconducting, metallic or semiconducting quantum rings [7,8] can [9], at least in principle, serve as a physical realization of qubits [9]. Effective controllability [10,11,12] and robustness against decoherence is a common feature shared by all groups of devices desired for realistic implementations. Quantum devices operating at a nano- and meso-scale working at a border of material science and quantum optics [13] can also serve as highly sensitive tools detecting subtle and non-classical features of quantum systems—with an entanglement [14,15] as the most often invoked example—utilized in quantum communication and quantum measurement process [16]. Among most peculiar features present at a nano-scale are (persistent) currents flowing in quantum rings (both superconducting and metallic [8]) which, due to non-trivial topology of multiply connected samples, are known to be highly sensitive and controllable via an external magnetic fields, both static [17,18,19] and time-dependent [20,21,22,23]. Properties of currents flowing in nanorings result from details of a material used for their construction and a concomitance of various noise sources [24] making their realistic and credible description highly non-trivial [25]. There are numerous studies relating microscopic description [26] of multi-particle low dimensional nanosystems to transport properties of conducting electrons encoded in Hamiltonian description including inter-particle interaction [27,28,29,30] and a highly non-trivial relation between transport properties in nanorings and various noise sources unavoidable affecting small samples [24].

Properties of persistent currents in a presence of a non-classical magnetic flux flowing both in superconducting and non-superconducting rings reflect many non-classical properties of magnetic flux treading the sample and modifying a phase of electrons. Carefully prepared non-classical electromagnetic fields applicable in quantum information processing [31,32,33,34,35] are shown to modify properties of currents flowing in nanosystems [20,36,37,38,39,40] (and Ref. [41] for detailed review) under *external field approximation* [36] assumed. External field approximation introduced in Ref. [36] is an extremely useful and comfortable kind of mean field approach neglecting, however, back-influence of nanodevice on properties of non-classical control. In this approximation the control field is assumed strong enough to maintain its state yet continuously affecting the nanosystem. Despite its usefulness, the external field approximation suffers from natural limitations: under this approximation a driving field affects the nanosystem being itself not modified. In other words: in the external field approximation a non-classical control is a truly *open-loop control* as it neglects interaction-induced feedback of a nanosystem coupled to the non-classical driving field. As a figure of merit one calculates the expectation values of local observables (e.g., currents flowing in a nanoring) by tracing (averaging) with respect to a state of a driving field *not* affected by a presence of the nanosystem. Let us emphasise that—contrary to a microscopic modelling which is going to be proposed here—such an approximate approach does not allow to investigate entanglement of the nanosystem and the driving non-classical field.

Our objective is to extend and supplement the previous studies [20,36,37,38,39,40,41] and go beyond the external field approximation. In analogy to phenomenological model for SQUIDs [42] or metallic ring [40] simplifying essentially many-body problem to a pseudo-spin (qubit) approximation, we propose fully microscopic many-body model of a nanoring carrying current of spin-less electrons. Neglecting spin degree of freedom we avoid the corresponding spin-boson interaction of the Jaynes–Cummings or Rabi type [16] between electrons in the nanoring and a non-classical driving fields. Consequently, the considered fermion–boson interaction is of the type studied previously in the external field approximation. However, contrary to the previous studies, we can—and we do—investigate entanglement of a nanoring and a driving non-classical field and, in particular, its relation to a previously neglected fermion–fermion interaction in the nanoring. Using the microscopic model we complement previous studies of nanorings undergoing a non-classical control and utilizing the external field approximation [38,39], pseudo-spin [9,40] or a semi-classical description [20].

Currents flowing in nanorings can exhibit non-trivial properties [24,43] under an external control. We consider a non-classical control of particles accommodating the ring by a single mode one-dimensional bosonic field (non-classical magnetic flux) coupled via a hopping part (Peierls phase) of the system Hamiltonian. We analyse a role played by non-local fermion–fermion interaction of a Coulomb type. Working beyond external field approximation we analyse interaction-induced creation of entanglement between fermions in the nanoring and the bosonic mode of electromagnetic field. We show that in an absence of inter-particle interaction (i.e., for a free electron model) particles in the ring the non-classical field and the ring remain unentangled despite non-trivial field-induced modification of current flowing in the nanoring. It holds true, however, provided that there is no source of dissipation in the system. This result confirms credibility of the external field approximation for currents in ring-shaped devices accommodating non-interacting and decoherence-free particles.

Our work is organized as follows: In Section 2 (Materials and Methods) a model of a nanoring controlled by a non-classical electromagnetic field is formulated. In Section 3 (Results) results of numerical analysis of the previously formulated model are presented both for unitary, decoherence-free, system and in a presence of Markovian dissipation affecting fermions in the nanoring. Finally, in Section 4 (Discussion) we discuss and summarize results of the work.

## 2. Materials and Methods

We propose a simple model of a quantum *L*–site fermionic system in a ring threaded by a line of magnetic flux Φ. We apply theoretical units $k_{B}=\hbar =c=1$ to simplify subsequent formulas, cf. Appendix B of Ref. [36] for a useful ’dictionary’ expressing standard units in terms of the theoretical units. In particular, for the theoretical units the electric charge used below is a dimensionless quantity reading $e=\sqrt{4\pi /137}$ and all the standard units can be expressed in terms of powers of electronvolts eV [36]. Let us note, however, that none of the (numerical) results of this work presented in next Sections depends on the applied system of units since they are presented as *ratios* of dimensional quantities (e.g., currents) or the quantities which are dimensionless by definition such as linear entropy. In particular, time units are scaled with respect to frequency of non-classical control field which is specified below.

The system is described by a Hamiltonian

(1) $$\begin{array}{ccc} {\hat{H}}_{0} & = & {\hat{H}}_{J}+{\hat{H}}_{U} \end{array}$$

consisting of a kinetic (‘hopping’) part ${\hat{H}}_{J}$ [26] and a part ${\hat{H}}_{U}$ describing a fermion–fermion interaction. For a purely classical magnetic flux Φ and spin-less fermions the hopping term of a strength *J* reads as follows [26]

(2) $$\begin{array}{ccc} {\hat{H}}_{J}\left(\phi \right) & = & -J\sum_{i}^{L}\left(e^{i\phi }{\hat{c}}_{i+1}^{†}{\hat{c}}_{i}+h.c.\right) \end{array}$$

where ${\hat{c}}_{i},{\hat{c}}_{i}^{†}$ are fermionic annihilation and creation operators at a site i∈[1,…,L] and h.c. denotes hermitian conjugate. The interaction part ${\hat{H}}_{U}$ is assumed to be of a Coulomb type, i.e., it describes interaction of charged particles with an amplitude *U*:

(3) $$\begin{array}{ccc} {\hat{H}}_{U} & = & U\sum_{i\neq j}^{L}\frac{{\hat{n}}_{i}{\hat{n}}_{j}}{d(i,j)} \end{array}$$

where ${\hat{n}}_{i}={\hat{c}}_{i}^{†}{\hat{c}}_{i}$ and d(·) is a distance (a number of sites) separating *i*–th and *j*–th site in the nanoring. Note the phase $\phi =2\pi \Phi /(L\phi_{0})$ which is related to magnetic flux Φ and the (dimensionless for the applied theoretical units) flux quantum $\phi_{0}=h/e=\sqrt{137\pi }$. As the fermions which we consider are spin-less, the magnetic field modifies the hopping part of the Hamiltonian Equation (1) only. The Peierls factor $e^{i\phi }$ affects also transport properties of fermions described by a current operator [26]

(4) $$\begin{array}{ccc} \hat{I}\left(\phi \right) & = & iI_{0}\sum_{i}\left(e^{i\phi }{\hat{c}}_{i+1}^{†}{\hat{c}}_{i}-h.c.\right) \end{array}$$

with an amplitude $I_{0}=2\pi J/\left(L\phi_{0}\right)$. Let us recall that a static magnetic flux Φ threading the ring results in *persistent current* [7,8], i.e., a non-dissipative direct component of current flowing in a long time limit in non-superconducting rings [21,23].

For a monochromatic non-classical field of frequency ω operating at low temperature T≪ω [41,44] a vector potential $\hat{A}$ and an electric field $\hat{E}$ are dual quantum variables [44]. The corresponding operators of a magnetic flux $\hat{\varphi }$ and an electromotive force ${\hat{V}}_{EMF}$ are given [41] by integrals $\hat{\varphi }=\oint_{C}\hat{A}dx$ and, respectively, ${\hat{V}}_{EMF}=\oint_{C}\hat{E}dx$ where the integration contour *C* is small with respect to a size of of the ring. A non-classical electromagnetic field, as an essentially bosonic system, allows for the Heisenberg–Weyl representation of the field operators $\hat{A},\hat{E}$ or $\hat{\varphi },{\hat{V}}_{EMF}$ in terms of bosonic annihilation $\hat{a}=\left(\hat{\varphi }+i\omega^{-1}{\hat{V}}_{EMF}\right)/\left(2\sqrt{\xi }\right)$ and creation ${\hat{a}}^{†}=\left(\hat{\varphi }-i\omega^{-1}{\hat{V}}_{EMF}\right)/\left(2\sqrt{\xi }\right)$ operators satisfying $\left[\hat{a},{\hat{a}}^{†}\right]=\hat{1}$ with ξ related to the area of *C* [44].

We consider a composite system consisting of a nanoring accommodating charged but spin-less fermions threaded by a magnetic flux having—in addition to a static and classical $\phi_{c}=2\pi \Phi /\left(L\phi_{0}\right)$ part—a *non-classical* component

(5) $$\begin{array}{ccc} \hat{\phi } & = & \phi_{c}+\phi_{1}\left(\hat{a}+{\hat{a}}^{†}\right) \end{array}$$

of an amplitude $\phi_{1}=2\pi \xi /\left(\sqrt{2}L\phi_{0}\right)$. A Hamiltonian of the composite ring plus field system and a corresponding current operator are then given by:

(6) $$\begin{array}{ccc} \hat{H} & = & {\hat{H}}_{J}\left(\hat{\phi }\right)+{\hat{H}}_{U}+\omega {\hat{a}}^{†}\hat{a} \end{array}$$

and

(7) $$\begin{array}{ccc} \hat{I} & = & \hat{I}\left(\hat{\phi }\right) \end{array}$$

with $\hat{\phi }$ given in Equation (5) and $\hat{I}\left(\cdot \right)$ given in Equation (4). Let us emphasise that for the spin-less fermions considered here typical spin–boson terms (such as, e.g., Jaynes–Cummings or Rabi) in the Equation (6) do not occur.

The Hamiltonian of the ring–field composite system Equation (6) is of the Caldeira–Leggett form [45], i.e., it contains distinguishable parts corresponding to subsystems and describing inter-subsystem interaction, however, it is necessary to emphasise non-perturbative character of coupling given by phase factor present in ${\hat{H}}_{J}$. Hence, a time evolution of the system generated by Equation (6) both in a presence and absence of decoherence is further analyzed numerically using QuTip [46,47], a Python-based toolbox designed for quantum control and evolution with an essentially infinite bosonic space span{|0〉,|1〉,⋯} accommodating non-classical field mode truncated to $\mathrm{span}\{|0\rangle ,|1\rangle ,\cdots ,|N_{B}\rangle \}$ with $N_{B}=10$ sufficient for credible modelling. We assume the composite system initially prepared in a separable state

(8) $$\begin{array}{c} \hat{\rho }\left(0\right)=|E_{0}\rangle \langle E_{0}|⊗{\hat{\rho }}_{B}\left(0\right) \end{array}$$

where $|E_{0}\rangle$ is the lowest energy eigenstate of ${\hat{H}}_{0}\left(\phi_{c}\right)$ Equation (1) of *two* fermions in a L=6—site ring threaded by classical flux only and ${\hat{\rho }}_{B}\left(0\right)$ is an initial state of the non-classical flux threading the ring. In other words, the fermionic system (ring) being in its ground state becomes initially tensorized with an electromagnetic quantum control field. Further we consider non-classical electromagnetic field affecting nanoring in one of two classes of initial states. The first, consisting of number eigenstates

(9) $$\begin{array}{ccc} {\hat{\rho }}_{B}^{\left(1\right)}\left(0\right) & = & \left|N\rangle \langle N\right| \end{array}$$

is chosen due to the known non-classical properties [44] of its constituents. The second class of states consists of coherent states forming pairs of the celebrated Schrödinger cats:

(10) $$\begin{array}{ccc} {\hat{\rho }}_{B}^{\left(2\right)}\left(0\right) & = & \left|\pm \rangle \langle \pm |,\;\;\;\;\;|\pm \rangle =N(|z\rangle \pm |-z\rangle \right) \end{array}$$

where |z〉 denotes a coherent state and N is a normalization factor. It is hard to overestimate a role played by coherent states in quantum information processing [31,32,33,34,35] and, in particular, the states in Equation (10), as orthogonal and well distinguishable, are natural candidates for logical qubits. Further we show that currents in nanorings can serve as an auxiliary quantifier for their detection.

Fragile nanosytems are subjected to decoherence and noise which often significantly affect their transport properties Ref. [24]. Decoherence effects originate from an interaction with the environment and result in a *non-unitary* corrections to a time-evolution. The system, treated as a whole with its its environment, is assumed to be closed and evolves unitarily, while its *reduced* evolution determines an (open) subsystem evolving according to a non-unitary operator $\hat{\rho }\left(t_{j}\right)=\Gamma \left(t_{j},t_{i}\right)\hat{\rho }\left(t_{i}\right)$ where $\hat{\rho }\left(t_{i}\right)$ is the reduced (with respect to the environment) density matrix of the time-evolving system [48]. The operator Γ must not be arbitrary–the transformation should be (at least) completely positive [48,49,50] and satisfying the semi-group property: $\Gamma \left(t_{2},t_{0}\right)=\Gamma \left(t_{2},t_{1}\right)\Gamma \left(t_{1},t_{0}\right)$. In particular, for $\Gamma \left(t_{j},t_{i}\right)=\Gamma \left(t_{j}-t_{i}\right)$ the time evolution is Markovian. Such conditions are fulfilled by the density matrix $\hat{\rho }\left(t\right)$ obeying a Markovian master Equation [48]:

(11) $$\begin{array}{c} \frac{d}{dt}\hat{\rho }=-i\left[\hat{H},\hat{\rho }\right]+L\left[\hat{\rho }\right] \end{array}$$

where initially we assume $\hat{\rho }\left(0\right)$ given by Equation (8). The Hamiltonian part of Equation (11) is generated by $\hat{H}$ defined in Equation (6). The (non-Hamiltonian) Lindbladian part of the master equation Equation (11) is responsible for effects connected with dissipation and decoherence. The minimal requirement imposed to its form is to satisfy the Gorini–Kossakowski–Lindblad–Sudarshan conditions [45,48] granting complete positivity of time-evolution Equation (11). There are many, usually problem-dependent, forms of the L[·] superoperator credibly modelling dissipative quantum dynamics [51]. Here we apply phenomenological modelling an we assume that the particle-field composite Equation (6) undergoes completely positive [45] time evolution governed by a master equation

(12) $$\begin{array}{ccc} \frac{d}{dt}\hat{\rho }\left(t\right) & = & -i\left[\hat{H},\hat{\rho }\left(t\right)\right]+\frac{\gamma }{2}\sum_{n=1}^{L}\left[2{\hat{c}}_{n}\hat{\rho }\left(t\right){\hat{c}}_{n}^{†}-\hat{\rho }\left(t\right){\hat{c}}_{n}^{†}{\hat{c}}_{n}-{\hat{c}}_{n}^{†}{\hat{c}}_{n}\hat{\rho }\left(t\right)\right] \end{array}$$

where the dissipative part, having a phenomenological amplitude γ, represents decoherence affecting particles in the nanoring. In a low temperature limit T≪ω—suitable for non-classical properties of electromagnetic fields [41] considered here—dissipators containing bosonic operators $\hat{a},{\hat{a}}^{†}$ are omitted. From a physical perspective the master equation Equation (12) describes completely positive dynamics of the nanoring–field system with a decoherence of the same strength locally affecting each of *L* lattice sites in the nanoring. Let us emphasise that Markovian modelling applied in Equation (12) despite its simplicity finds broad and fruitful applications in studies of many-body models [52,53].

Interaction between subsystems of the nanoring–field composite Equation (6) generically results in an entanglement [14,50] of fermionic and bosonic degrees of freedom. Both its qualification and quantification, due to system’s dimensions and decoherence in Equation (12), remain non-trivial [14,15]. However, for γ→0 one arrives at a unitary limit considerably simplifying the task. We use two quantifiers to evaluate (bipartite) entanglement of the (fermionic) nanoring and (bosonic) non-classical field. The first, suitable for pure states [14,15] $\hat{\rho }\left(t\right)$, is a linear entropy:

(13) $$\begin{array}{ccc} S_{L} & = & \frac{N_{B}}{N_{B}-1}\left(1-Tr({\hat{\rho }}_{B}^{2})\right) \end{array}$$

of state ${\hat{\rho }}_{B}={Tr}_{F}\hat{\rho }$ reduced with respect to one of two subsystems, here chosen to be fermionic denoted by *F*. Linear entropy $S_{L}$ in Equation (13) is chosen to be normalized with respect to the (truncated) dimension $N_{B}$ of the bosonic space of non-classical field. A positive value of $S_{L}$ indicates mixedness of the reduced state $\hat{\rho }\left(B\right)$ and hence it is a hallmark of an entanglement of a pure state $\hat{\rho }$. Let us note that the entropic criterion $S_{L}>0$ cannot be effectively applied [14,15] to detect entanglement of generic mixed states appearing, e.g., in a system subjected to decoherence [45]. As a generic problem of detecting bipartite entanglement of *mixed* states so far remains unsolved [14] we limit our attention to entangled states which satisfy the Peres criterion [14] and are negative with respect to partial transposition (NPT entangled states). The NPT entanglement can be quantified by the negativity [15]

(14) $$\begin{array}{ccc} N & = & \frac{1}{2}\sum_{\lambda }\left(|\lambda |-\lambda \right) \end{array}$$

where λs are eigenvalues of a partially transposed ring–field state.

## 3. Results

**Figure 1:**
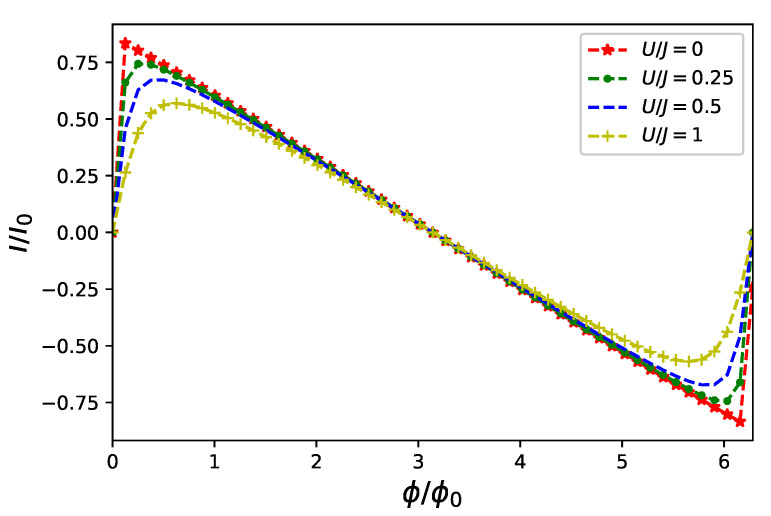
Persistent current flowing in a ring of L=6 sites accommodating two (spin-less) fermions interacting via Hamiltonian in Equation (1) for different values of fermion–fermion interaction *U* initially prepared in its ground state.

Persistent currents driven by a classical flux only depend on an amplitude of fermion–fermion interaction *U* Equation (3). In Figure 1, we present steady-state currents flowing in a ring of L=6 sites accommodating two particles initially prepared in a ground state (the lowest energy eigenstate) of Equation (1).

Results presented in Figure 1 re-confirm well known [8] role of the inter-carrier interaction modifying an amplitude of persistent current what is best visible for magnetic flux $\phi_{c}<\pi /2$ indicating a range of external control parameter Φ adequate for an effective enhancement of a role of Coulomb interaction in a system. As an effect of *U* is going to be further investigated, to enhance its visibility, we fix the steady classical flux in Equation (6) $\phi_{c}=\pi /4$.

### 3.1. Unitary Evolution

There are two mechanisms, except non-classical electromagnetic control, present in the model Equation (6) and considered in this work competitively affecting an amplitude of a current flowing in the nanoring. The first and most obvious is decoherence in Equation (12) which discussion is postponed to the next subsection. The second is a Coulomb-like inter-particle interaction Equation (3) which is going to be studied here in a formal unitary limit of Equation (12) for γ=0 for the nanoring controlled by a non-classical flux.

**Figure 2:**
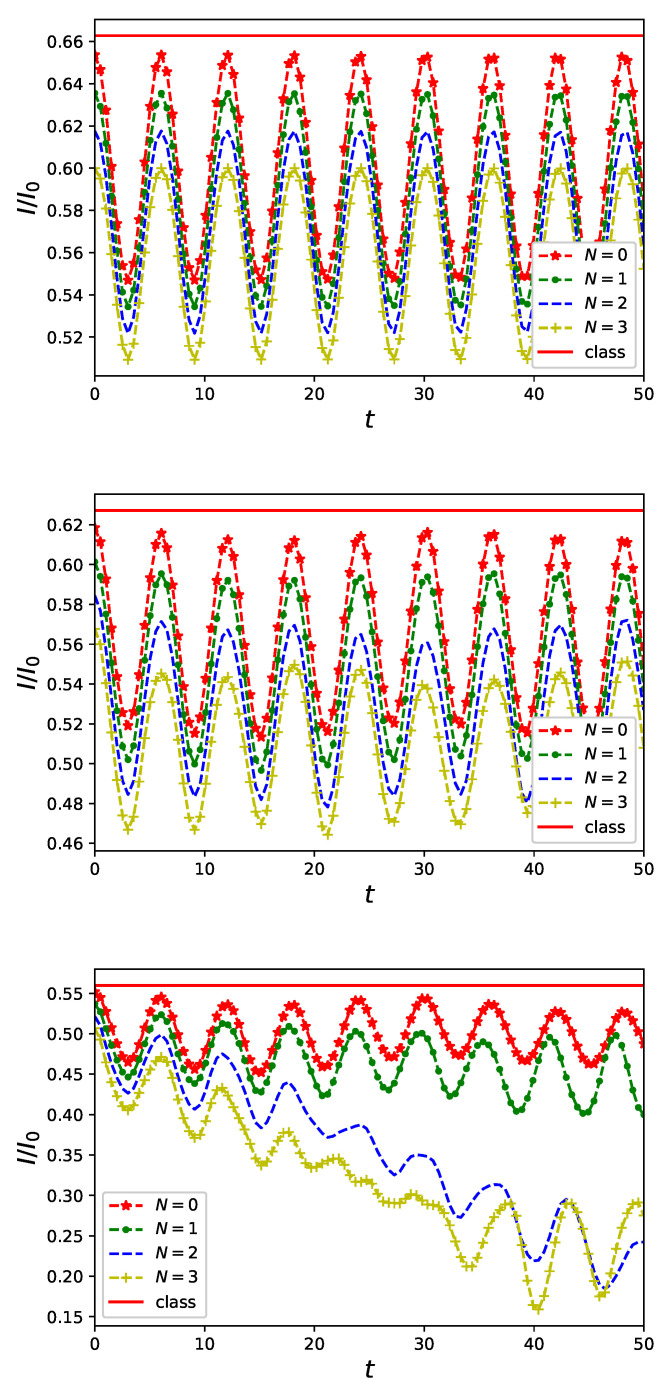
Current flowing in a quantum ring of L=6 sites accommodating two spin-less fermions in a presence of non-classical flux in a number state |N〉 with (i) U/J=0 (**upper panel**), (ii) U/J=0.5 (**central panel**) and (iii) U/J=1 (**lower panel**). Solid lines denoted by ‘class’ indicate (steady) value of persistent current in an absence of non-classical control.

In Figure 2, we present expectation values the current operator Equation (7) for the electromagnetic field initially in a number state Equation (9) for different values of *U*. An upper panel of Figure 2 where the current of non-interacting particles U=0 is plotted, indicates that non-classical driving results in a lowering of the current amplitude which, in particular, occurs also for non-classical fields in the vacuum state |N=0〉. Moreover, non-classical control of nanorings results in periodic current characteristics in contrast to stationary and steady persistent current flowing in a presence of a classical flux $\phi_{c}$ only. In an absence of particle–particle interaction (U=0) current characteristics depends on the initial number state of the flux in nothing but an amplitude of the oscillations. It is not the case any more for interacting particles carrying current in the nanoring as can be inferred from central and bottom panels of Figure 2. For U/J=0.5 (bottom panel of Figure 2) both an amplitude and periodicity of the current characteristics becomes a hallmark of larger values of *N* in an initial state Equation (9) |N〉 of the driving flux. Let us note that potential usefulness of currents flowing in nanorings in discrimination of a state of non-classical radiation becomes limited for large values of a fermion–fermion interaction *U* as it can be inferred from a bottom panel of Figure 2 exhibiting intersection of graphs for different states |N〉 of the controlling field. To overcome this drawback it is necessary to investigate currents in a tailored, relatively short, time regime.

**Figure 3:**
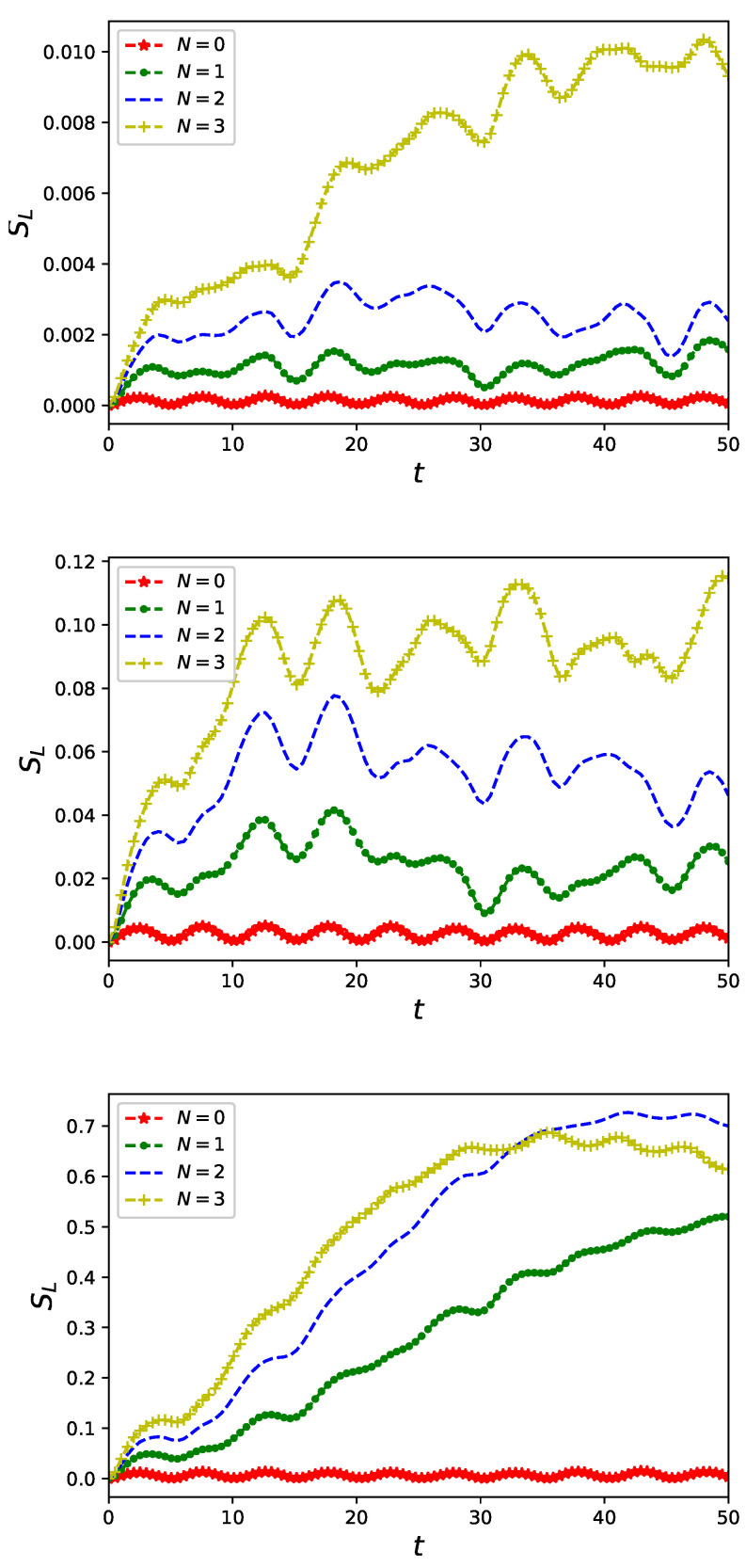
Linear entropy Equation (13) of a reduced (with respect to fermionic degrees of freedom) state of a system of quantum ring of L=6 sites accommodating two spin-less fermions interacting with a single-mode non-classical flux in a number state |N〉 with (i) U/J=0.1 (**upper panel**), (ii) U/J=0.5 (**central panel**) and (iii) U/J=1 (**lower panel**).

Linear entropy Equation (13) quantifying entanglement of fermionic and bosonic degrees of freedom of a composite system consisting of a nanoring and controlling non-classical field is presented in Figure 3 for U/J=0.1 (upper panel), U/J=0.5 (central panel) and U/J=1 (bottom panel). A feature which is peculiar and needs to be emphasised is that in an absence of interaction of particles the nanoring and the non-classical field, initially separated Equation (8) stay *unentangled*, i.e., for U=0 linear entropy vanishes $S_{L}=0$ as time flows confirming validity external field approximation applied in a series of papers [20,36,37,38,39,40,41,54] for a free electron model of conduction. Let us also note the intersection of graphs in the bottom panel of Figure 3 analogous to previously presented intersection of current characteristics.

**Figure 4:**
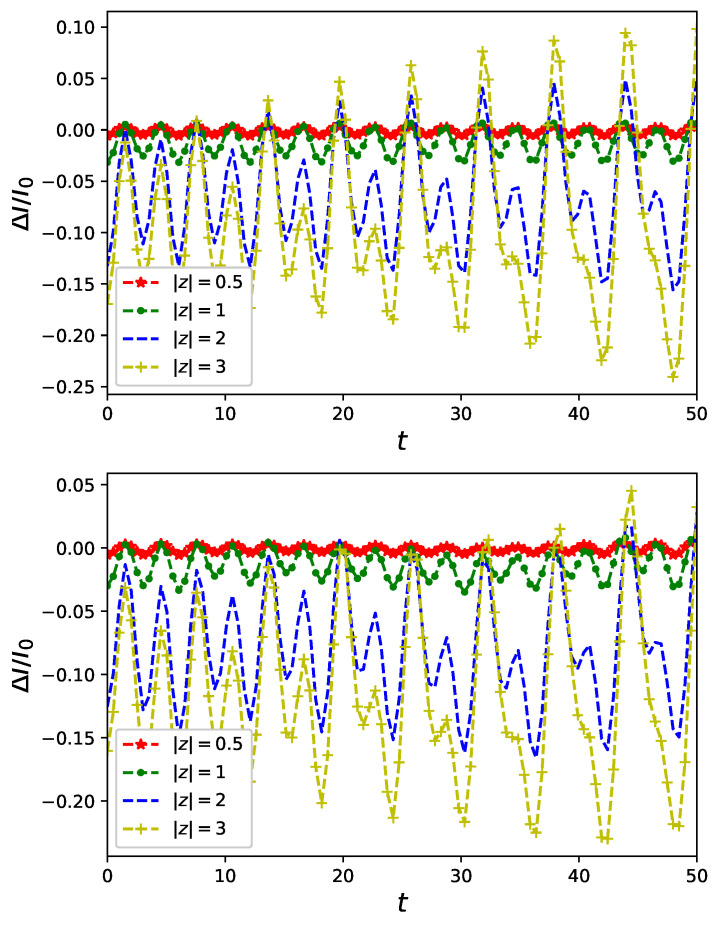

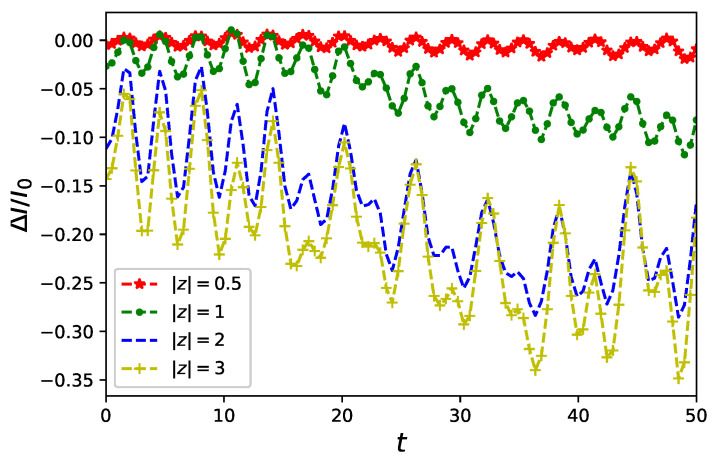
Difference of currents flowing in a quantum ring of L=6 sites accommodating two spin-less fermions in a presence of non-classical flux initially in cat states Equation (10) for different values of coherent state ||z|〉: (i) U/J=0 (**upper panel**), (ii) U/J=0.5 (**central panel**) and (iii) U/J=1 (**lower panel**).

There is a potential ability of currents flowing in nanoring to serve as an auxiliary tool for distinguishing logical qubits Equation (10) in quantum communication. Using a hypothetical device utilizing *identical* rings of possibly unknown parameters to classify incoming logical qubits into one of the two types Equation (10) it is sufficient to know that one among two logical qubits induces *larger* current flowing in the nanonrings. To achieve this goal—i.e., to discriminate the logical qubits exiting communication channel—it is not necessary to know a particular and precise value of a current in the nanoring corresponding to a given logical qubit. It is sufficient to know that different logical qubits cause different reaction of the currents used as ’detectors’. Such a two-valued reaction (corresponding to a larger or smaller current) could be (at least in principle) translated into classical bits of information. In other words, it is enough to analyse a difference of currents ΔI rather than their particular values provided that the difference, quantifying different response of the nanorings to a particular logical qubit, is of a significant magnitude. Such an approach could be beneficial for nanorings made of materials requiring sophisticated modelling [24] leading to an often associated doubtful credibility of theoretical predictions of concrete values of currents. There is a difference ΔI of currents in a presence of non-classical flux prepared in the state $\rho_{f}^{2}\left(0\right)=\left|+\rangle \langle +\right|$ and $\rho_{f}^{2}\left(0\right)=\left|-\rangle \langle -\right|$ presented in Figure 4. It is plotted for different values of U/J=0,0.5,1 for upper, central and bottom panels, respectively. Let us note that for sufficiently large values of *z* in Equation (10) the difference between currents corresponding to different logical qubits become also significant. Let us emphasise that a sign of ΔI changes in time as presented in the bottom panel of Figure 4. The reversal of the sign of the current difference is an obstruction for using nanorings as detectors as it prevents to uniquely assign signals (corresponding either to larger or to smaller currents in the ring) to a particular quantum-logical value of a controlling field. For a potentially credible discrimination of the two logical qubits Equation (10) with help of nanorings time of interaction between nanorings and the field needs to be balanced (neither to short nor to long) to reach satisfactory amplitude of ΔI without approaching the sign reversal.

**Figure 5:**
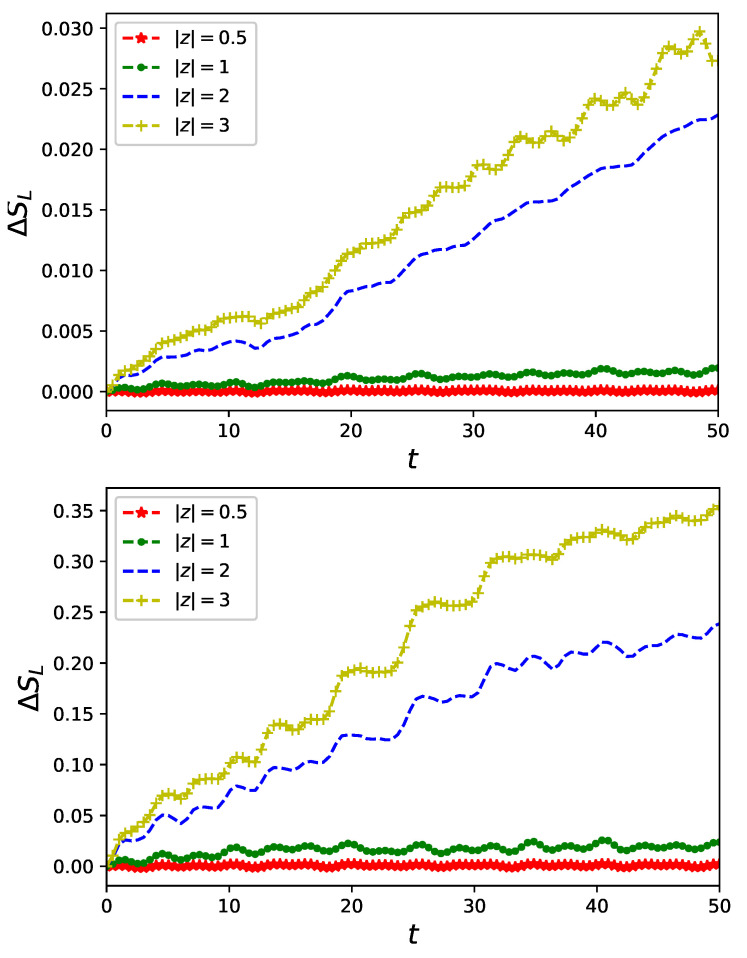

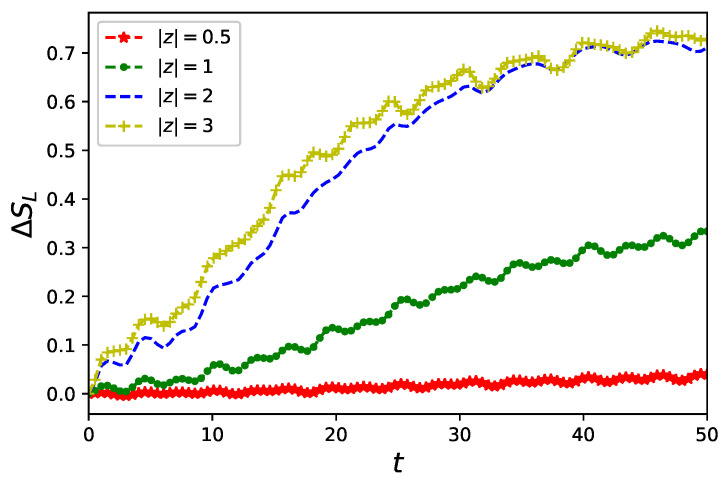
Difference of linear entropy Equation (13) of a reduced (with respect to fermionic degrees of freedom) state of a system of quantum ring of L=6 sites accommodating two spin-less fermions interacting with a single-mode non-classical flux initially in Cat States Equation (10) for different values of coherent state ||z|〉: with (i) U/J=0.1 (**upper panel**), (ii) U/J=0.5 (**central panel**) and (iii) U/J=1 (**lower panel**).

The corresponding difference in linear entropy Equation (13) given in Figure 5 indicates growing entanglement difference with increasing *U*. This feature indicates that a detection of different logical qubits corresponding to the Schrödinger cats Equation (10) with help of nanorings is associated with a different degree of entanglement between message carrying qubits and the detector.

### 3.2. Markovian Dissipation

**Figure 6:**
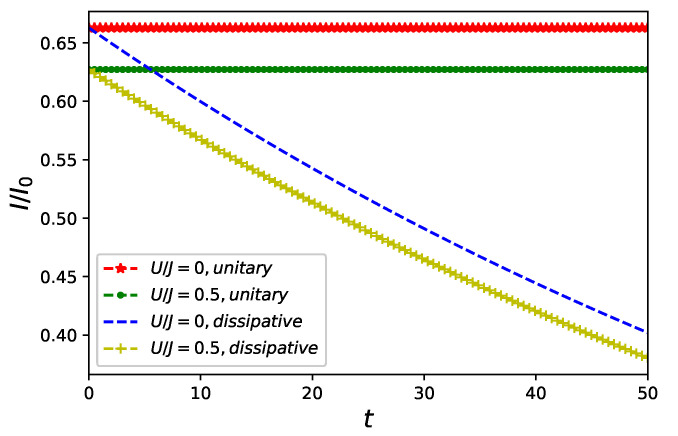

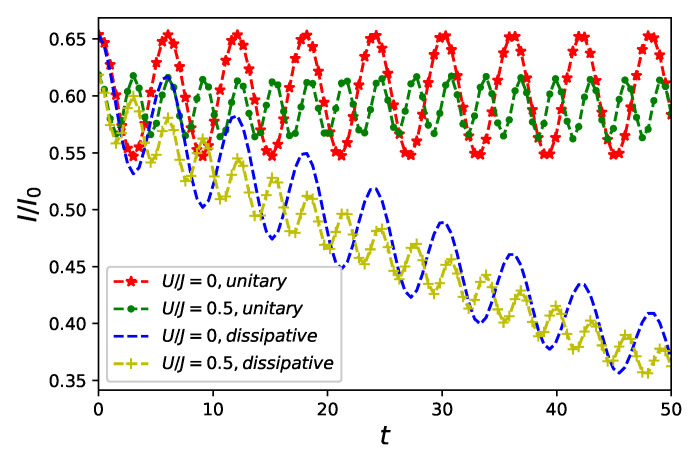
Current flowing in a quantum ring of L=6 sites accommodating two spin-less fermions in a presence of classical flux $\phi_{c}=\pi /4$ only (**upper panel**) and in a presence of non-classical flux in a vacuum state |N=0〉 (**lower panel**) for unitary γ=0 or dissipative γ=0.1 evolution generated by in Equation (12) without (U=0) and with (U=0.5) inter-particle interaction Equation (3).

In this subsection an effect of weak Markovian dissipation affecting particles in the nanoring Equation (12) is presented. The aim is to verify to what extent *U*–dependence of $S_{L}$ and in particular unusual perpetual separation of free (U=0) electrons and the non-classical driving remains valid in a system described by a dissipative model mimicking realistic conditions in which nanorings do operate. In an absence of non-classical control the dissipation results in lowering of a current amplitude both in a presence and in an absence of inter-particle interaction as it can be inferred from an upper panel of Figure 6. Qualitatively similar behaviour but supplemented with periodic oscillations occurs for nanorings threaded by a non-classical flux initially in its vacuum state |N=0〉 as presented in Figure 6 (lower panel).

**Figure 7:**
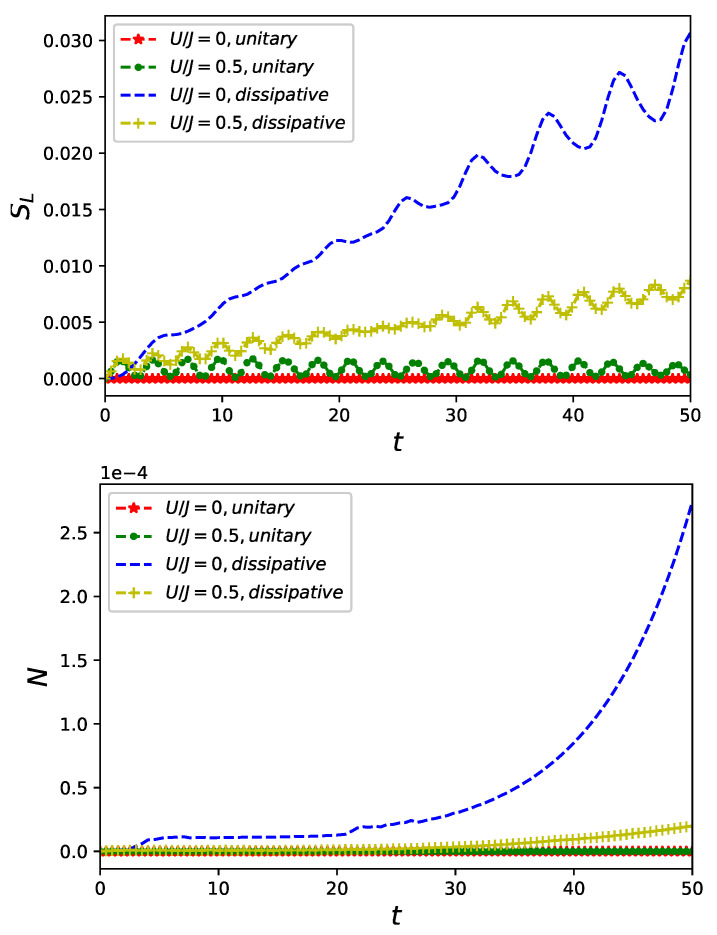
Linear entropy Equation (13) (**upper panel**) and negativity Equation (14) (**lower panel**) of a state of a system of quantum ring of L=6 sites accommodating two spin-less fermions interacting with a single-mode non-classical initially prepared in a vacuum state |N=0〉 with and without inter-particle interaction Equation (3) for unitary γ=0 or dissipative γ=0.1 evolution generated by in Equation (12) for unitary γ=0 or dissipative γ=0.1 evolution generated by in Equation (12).

As a time evolution generated by Equation (12) is dissipative and in general entropy-increasing [45], linear entropy $S_{L}$, although not a proper entanglement quantifier, still serves as a natural measure of mixedness [1]. Let us note that in a presence of non-classical driving the linear entropy $S_{L}$ grows faster in an absence of fermion–fermion interaction U=0 as it is presented in an upper panel of Figure 7. Similar behaviour is present also for an NPT entanglement of fermionic and bosonic degrees of freedom quantified by negativity and plotted in a lower panel of Figure 7. Let us notice that the non-classical driving induces NPT entanglement also in an absence of inter-particle interaction in the dissipative nanoring.

## 4. Discussion

Our research on the model proposed in Equation (6) of a quantum nanoring controlled by non-classical electromagnetic fields was focused on two aspects of time evolution governed by a Markovian master equation Equation (12): the first is a fermionic current flowing in the ring and the second is the fermion–boson entanglement originating from the ring–field coupling Equation (6). We investigated properties of currents flowing in the ring for selected states of controlling flux threading the ring. We limited our attention to number states of radiation of a well known non-classical properties [41]. We showed that the non-classical driving modifies current characteristics from stationary, typical for persistent currents [8] (cf. Figure 1), to oscillatory in time with a period depending on a frequency ω Equation (6) of the non-classical field as presented in Figure 2. Due to an essentially non-perturbative character of the ring–field coupling Equation (2) we performed numerical calculations to find time evolving state of the composite system Equation (12) taking into account possible Coulomb-like interaction between particles accommodating the nanoring Equation (3). We showed that, except expected lowering of a current amplitude (cf. Figure 2), inter-particle interaction strongly influences fermion–boson entanglement (cf. Figure 3) quantified (for pure states) by linear entropy Equation (13). In an absence of the interactions current carrying particles and non-classical control field, initially in a separable state, *remain unentangled* as time flows. At the same time, however, an effect of interaction is manifested in current time characteristics. This observation which is the main result of our present studies additionally justifies applicability of an external field approximation utilized in previous studies [20,36,37,38,39,40,41,54] for a free-electron model of conduction.

Sensitivity of currents flowing in nanorings can be utilized as an auxiliary tool for detecting properties of non-classical flux. We compare currents controlled by a pair of Schrödinger cat states Equation (10) which are natural candidates for logical qubits in applications of quantum information processing based upon coherent states [31,35]. We showed a different response of currents in nanorings driven by such states cf. Figure 4. This observation can, at least in principle, be useful in quantum communication protocols utilizing Schrödinger cats as ’letters’ of a transmitted message. Observing a value of a difference of currents flowing in nanorings to discriminate one of two letters of an alphabet formed by the two Schrödinger cats. Potential effectiveness of such a procedure depends, as it is presented in the previous Section, not only on the state |z〉 constructing the Schrödinger cats but also on a duration of a nanoring–field interaction. It is also shown cf. Figure 5 that an entanglement induced by the interaction can be enhanced by a proper choice of parameters of initial states of non-classical control Equation (10).

The ring–field entanglement is generated for U≠0 in Equation (3) or—it is the second main conclusion of our work—by a presence of decoherence affecting (as it is assumed in our work) particles accommodating nanoring. An amplitude of currents flowing in a dissipative nanoring becomes damped in comparison to a unitary evolving systems cf. Figure 6. To quantify ring–field entanglement for dissipative ring–field system we utilize negativity Equation (14) allowing to detect NPT entanglement as presented Figure 7. We thus infer an additional requirement for a credibility of an external field approximation: decoherence, if present in a system, should be weak. A simultaneous presence of decoherence and an inter-particle many-body interaction does not act ’additively’ in enhancing NPT entanglement: simultaneous presence of both decoherence and an inter-particle interaction does not result in entanglement (quantified by the negativity) being larger than it is in a presence decoherence only. It is also the case for ’mixedness’ of reduced state qualified by linear entropy cf. Figure 7.

Nano-devices form a bridge between classical world of information engineering and deeply microscopic stage of quantum phenomena [2,3]. Nanorings, due to their non-standard properties originating from non-trivial doubly-connected topology, are natural candidates for implementations of quantum information processing: from qubits, via quantum communication up to a quantum measurement and control. Microscopic description of nanorings threaded by non-classical flux indicates non-trivial dynamic properties deserving for a multi-perspective investigations. A research direction based upon the nanoring-field model formulated in this work is one of the proposals.

## Data Availability

Not applicable.
